# The Longitudinal Force Measurement of CWR Tracks with Hetero-Cladding FBG Sensors: A Proof of Concept

**DOI:** 10.3390/s16122184

**Published:** 2016-12-18

**Authors:** Li-Yang Shao, Meng Zhang, Kaize Xie, Xinpu Zhang, Ping Wang, Lianshan Yan

**Affiliations:** 1 MOE Key Laboratory of High-Speed Railway Engineering, Southwest Jiaotong University, Chengdu 610031, China; kzxie@my.swjtu.edu.cn; 2 School of Information Science and Technology, Southwest Jiaotong University, Chengdu 610031, China; mengzh@my.swjtu.edu.cn (M.Z.); xpzhang@home.swjtu.edu.cn (X.Z.); lsyan@home.swjtu.edu.cn (L.Y.)

**Keywords:** longitudinal additional force, hetero-cladding fiber Bragg grating, continuous welded rail

## Abstract

A new method has been proposed to accurately determine longitudinal additional force in continuous welded rail (CWR) on bridges via hetero-cladding fiber Bragg grating (HC-FBG) sensors. The HC-FBG sensor consists of two FBGs written in the same type of fiber but with different cladding diameters. The HC-FBGs have the same temperature sensitivity but different strain sensitivity because of the different areas of the cross section. The differential strain coefficient is defined as the relative wavelength differences of two FBGs with the change of applied longitudinal force. In the verification experiment in the lab, the HC-FBGs were attached on a section of rail model of which the material property is the same as that of rail on line. The temperature and differential strain sensitivity were calibrated using a universal testing machine. As shown by the test results, the linearity between the relative wavelength difference and the longitudinal additional force is greater than 0.9999. The differential strain sensitivity is 4.85 × 10^−6^/N. Moreover, the relative wavelength difference is not affected by the temperature change. Compared to the theoretical results, the accumulated error is controlled within 5.0%.

## 1. Introduction

The high speed railway has become one of the safest and most popular modes of transportation. With global railway modernization, the total kilometrage of continuous welded rail (CWR) has rapidly increased up to over 500,000 km. The using of the new type of track structure enhances the strength and stability of CWR. However, the longitudinal force in CWR will increase significantly in some special zones with large temperature differences, small radii, or large span bridges because of the thermal expansion effect or the intensified track–bridge interaction. The increased force may lead to rail buckling deformation or rail breakage; therefore, derailment accidents may occur and cause significant casualties and economic losses [1,2,3]. Thus, longitudinal force monitoring of CWR is essential for ensuring the riding safety of railway vehicles, especially in railway bridges.

The fiber Bragg grating (FBG) sensor has been widely used in CWR monitoring because of its long service life, high precision, anti-electromagnetic interference, suitable for harsh condition, etc. [4,5,6,7,8,9]. Several review papers have introduced the application of FBG sensors in the railway industry [10]. They can be divided into two categories: dynamic measurements and static measurements. Most reported work focuses on evaluating and characterizing track dynamics data (vehicle position, speed and acceleration, wheel/rail forces, and track deformation) collected as trains pass through sensor-armed sections. These data are primarily derived from wheel/rail load data provided by the track environment. Yan et al. proposed an axle counting system based on matched FBG pairs [11]. Wei et al. demonstrated the feasibility of detecting wheel tread deformation by the residual vibration index when the vehicle travels over the FBG strain sensor [12]. Tam’s group has developed a real-time monitoring system on track and vehicle based on the relationship between the rail load and the rail deformation [4]. Catalano et al. presented an intrusion detection system for the protection of railway assets using FBG sensors [13]. Buggy identified the features indicative of changes in the condition of the critical track components such as fishplates, switchblades, and stretcher bars in the UK [14]. These investigations are all based on dynamic strain measurement. They are quite different from the long-term monitoring of longitudinal force on track, which is a static measurement. Wang et al. tested the longitudinal force of the rail based on a 3D beam model at the breathing area of CWR [15]. Dai provided the installation and sealing design for a FBG sensor for monitoring rail temperature force, the relative displacement of multilayered structures, and the closure status of turnout [16]. The longitudinal additional force is primary generated from the deformation of the bridge, and it will transfer to the CWR via the interaction between bridge and rail, and accumulate in rail. The longitudinal additional force is directly related to the strength and stability of the rail and plays an important role in the design of the CWR on the bridge. Besides the longitudinal additional force, the temperature force caused by the change of rail temperature is also reserved in rail. Due to the influence of the temperature compensation, the previous continuous testing method cannot distinguish the longitudinal additional force and the temperature force directly.

In this work, a new grating structure, a hetero-cladding fiber Bragger grating, has been proposed to measure the longitudinal additional force of CWR on bridges. Due to the identical temperature sensitivity and differential strain sensitivity, the longitudinal additional force can be measured by the relative wavelength differences of two FBGs. Moreover, the principle of hetero-cladding FBG sensors has been verified through indoor tests. The experimental results reveal that the relative wavelength difference changed linearly as the longitudinal additional force and the differential strain sensitivity is 4.85 × 10^−6^ *ε*^−1^. Moreover, the relative wavelength difference is not affected by the temperature change. Compared to theoretical results, the relative error between test and the theoretical value is less than 5.0%.

## 2. Principle

**Figure 1:**
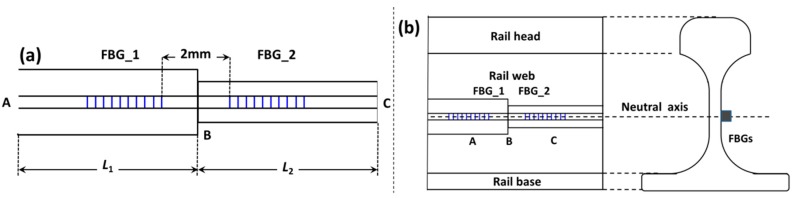
(**a**) Schematic diagram of hetero-cladding FBG sensors; (**b**) sensor installation diagram.

The structure of the hetero-cladding FBG sensors is schematically shown in Figure 1a. It consists of two FBGs with different cladding thicknesses separated by a fiber that is 2 mm long. The FBGs were fabricated in a singlemode fiber using the phase mask-based FBG writing technology, and the central wavelength of FBG_1 and FBG_2 are different. The hetero-cladding structure is obtained via the chemical etching method. FBGs with different cladding thicknesses can be fabricated by controlling the etching time. In practical applications, in order to avoid the influence of the wheel concentrated load, Points A and B (as shown in Figure 1a) should be fixed along the neutral axis of the rail between two fasteners, as illustrated in Figure 1b. In addition, during the sensor installation, the pre-stress must be applied to tense the fiber between Points A and C when the temperature changes.

In a FBG, the core mode can be coupled to the backpropagating core mode. The Bragg wavelength is determined by the phase match condition. The Bragg wavelength for core–core mode coupling is given by

(1) $$\lambda =2n_{eff}\Lambda$$

where *n_eff_* is the effective index of the core mode, and *Λ* is the period of the FBG. When the FBG is subjected to external perturbations, the wavelengths drift (Δ*λ*) induced by the temperature changes (Δ*T*) and strain changes (Δ*ε*) is given by [7]

(2) Δλλ=KεΔε+KTΔT

where *K_ε_* is strain sensitivity coefficient, which depend on the Poisson ratio and effective refractive index of core and cladding. *K*_T_ = ζ + α is the temperature sensitivity coefficient, which is related to the thermal expansion α and thermo-optic coefficient ζ. Since the temperature sensitivity coefficient is determined by the fiber materials, the temperature sensitivity coefficients of the two FBGs are equivalent. Δ*ε* and Δ*T* are the variations of temperature and strain, respectively. When the temperature changes, the FBG will deform due to thermal expansion, but the thermal strain cannot be considered a fraction of *Kε*. It should be attributed to temperature terms.

Assuming that the strain sensitivity coefficients of the two FBGs are *Kε*_1_ and *Kε*_2_, the cross-sectional areas are *A*_1_ and *A*_2_, respectively. The strain change of the CWR induced by bridge expansion is Δ*ε*_f_, and the temperature variation is Δ*t*. Moreover, due to the same material, the temperature sensitivity coefficient *K*_T_ and the modulus of elasticity *E* of FBGs are identical. In order to simplify the derivation, we assumed that *K*_ε2_ = *k*_ε_*K*_ε1_, *A*_2_ = *k*_a_*A*_1_, and *L*_2_ = *k*_l_*L*_1_. When the FBG is only subjected to temperature perturbation, the FBG will deform because of thermal expansion. The loss of prestress is Δ*F_y_* while the temperature variation is Δ*t*. According to the deformational compatibility condition [17],

(3) $$\frac{\Delta F_{y}L_{1}}{EA_{1}}+\frac{\Delta F_{y}L_{2}}{EA_{2}}=\alpha \Delta t\left(L_{1}+L_{2}\right)$$

where *L*_1_ and *L*_2_ are the distances between Points A and B and between Points B and C, respectively. The loss of prestress Δ*F_y_* can be expressed as

(4) $$\Delta F_{y}=E\alpha \Delta tk_{a}A_{1}\frac{1+k_{l}}{k_{a}+k_{l}}.$$

Therefore, the thermal-induced strain of the two FBGs can be given by

(5) $$\begin{array}{l} \varepsilon_{1t}=-\frac{\alpha \Delta tk_{a}\left(1+k_{l}\right)}{k_{a}+k_{l}}\\ \varepsilon_{2t}=-\frac{\alpha \Delta t\left(1+k_{l}\right)}{k_{a}+k_{l}} \end{array}.$$

When the strain applied to CWR is Δ*ε*_f_, two FBGs are subjected to external stress, which will induce additional strain. According to the force balance and deformational compatibility condition,

(6) $$\left\{ \begin{array}{l} EA_{1}\varepsilon_{1f}=EA_{2}\varepsilon_{2f}\\ \varepsilon_{1f}L_{1}+\varepsilon_{2f}L_{2}=\varepsilon_{f}\left(L_{1}+L_{2}\right) \end{array}\right..$$

As the longitudinal additional force is applied to rail, the strain evolution of the two FBGs is

(7) $$\begin{array}{l} \varepsilon_{1f}=\frac{L_{1}+L_{2}}{L_{1}\left(1+\frac{A_{1}L_{2}}{A_{2}L_{1}}\right)}\varepsilon_{f}=\frac{k_{a}\left(1+k_{l}\right)}{k_{a}+k_{l}}\varepsilon_{f}\\ \varepsilon_{2f}=\frac{l_{1}+l_{2}}{l_{2}\left(1+\frac{A_{2}l_{1}}{A_{1}l_{2}}\right)}\varepsilon_{f}=\frac{1+k_{l}}{k_{a}+k_{l}}\varepsilon_{f} \end{array}.$$

According to Equation (2) and simultaneously considering the evolution of temperature and strain,

(8) $$\begin{array}{l} \Delta \lambda_{1}/\lambda_{1}=K_{\varepsilon 1}\left(\varepsilon_{f1}+\varepsilon_{t1}\right)+K_{T}\Delta t=K_{\varepsilon 1}\frac{k_{a}\left(1+k_{l}\right)}{k_{a}+k_{l}}\left(\varepsilon_{f}-\alpha \Delta t\right)+K_{T}\Delta t\\ \Delta \lambda_{2}/\lambda_{2}=K_{\varepsilon 2}\left(\varepsilon_{f2}+\varepsilon_{t2}\right)+K_{T}\Delta t=k_{\varepsilon }K_{\varepsilon 1}\frac{1+k_{l}}{k_{a}+k_{l}}\left(\varepsilon_{f}-\alpha \Delta t\right)+K_{T}\Delta t \end{array}.$$

and

(9) $$\begin{array}{l} \frac{\Delta \lambda_{1}}{\lambda_{1}}-\frac{\Delta \lambda_{2}}{\lambda_{2}}=K_{\varepsilon 1}\frac{\left(1+k_{l}\right)\left(k_{a}-k_{\varepsilon }\right)}{k_{a}+k_{l}}\left(\varepsilon_{f}-\alpha \Delta t\right)=K\left(\varepsilon_{f}-\alpha \Delta t\right)\\ F_{f}=E_{s}A_{s}\varepsilon_{f}=\frac{E_{s}A_{s}}{K}\left(\frac{\Delta \lambda_{1}}{\lambda_{1}}-\frac{\Delta \lambda_{2}}{\lambda_{2}}\right)+E_{s}A_{s}\alpha \Delta t \end{array},$$

where *K = K*_ε1_(1 + *k_l_*)(*k*_a_ − *k*_ε_)/(*k*_a_ − *k_l_*), and *E_s_* and *A_s_* are the modulus of elasticity and cross-sectional area of the rail, respectively. In order to ensure the accuracy of the test results, the difference between *k*_a_ and *k*_ε_ should be increased to avoid *K* close to 0. Additionally, since the thermal expansion coefficient of fiber is very small, the thermal effects on the longitudinal force measurement on rail would be negligible. Therefore, the longitudinal additional force can be simplified as follows:

(10) $$F_{f}=E_{s}A_{s}\varepsilon_{f}=\frac{E_{s}A_{s}}{K}\left(\frac{\Delta \lambda_{1}}{\lambda_{1}}-\frac{\Delta \lambda_{2}}{\lambda_{2}}\right).$$

In practical applications, the longitudinal additional force and temperature are simultaneously applied to rail, we can calculate the longitudinal additional force on rail by substituting the temperature measurement data into Equation (9).

## 3. Experimental Verification

To verify the proposed principle, we implemented experiments in the laboratory to demonstrate its feasibility. Firstly, we should investigate the temperature sensitivity coefficients of the two FBGs to determine the existence of precondition. Thus, *K* in Equation (9) or (10) should be obtained. Finally, the correctness of the test principle and the test method can be determined by comparing the theoretical and experimental results.

### 3.1. Test Apparatus

**Figure 2:**
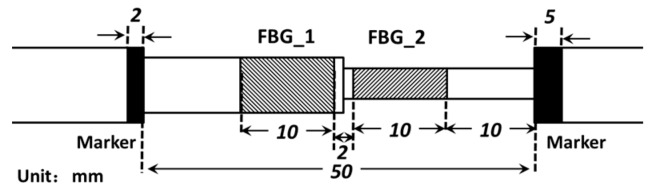
Schematic diagram of the geometric structure of the packaged sensor.

The FBGs used in experiment were fabricated with a SMF-28e fiber using a phase mask-based FBG writing technology. Moreover, the central wavelength of FBG_1 and FBG_2 are 1545 nm and 1544 nm, respectively. The hetero-cladding structure is obtained with the chemical etching method, and the FBGs with different cladding thicknesses can be controlled by adjusting the etch time. The geometric size and structure of the sensor is schematically shown in Figure 2. As shown in Figure 2, the length of the two FBGs and the separation between them are 10 mm and 2 mm, respectively, and the size of the whole packaged sensor is about 50 mm. In order to facilitate the installation of the packaged sensor, we set location markers at both ends of the packaged structure.

In this setup, the shift of the central wavelengths of the FBG sensors was measured by a single-channel FBG interrogator (SM130 from MOI Inc., Atlanta, GA, USA, a maximum sampling rate of 100 Hz and an accuracy of 1 pm). The permitted temperature variation range of the temperature control box (TCB) for the test is from −70 °C to 180 °C. The temperature control accuracy is ±0.5 °C. The maximum range of the universal testing machine in the test is 50 kN and the accuracy is 0.1 N. In the indoor test, the modulus of elasticity *E*, the thermal expansion coefficient, and the Poisson ratio of the rail material are 2.06 × 10^11^ Pa, 1.18 × 10^−5^/°C, and 0.3, respectively. The cross-sectional area of the rail model in the direction to be tested is 10.76 × 10^−4^ m^2^.

### 3.2. Test Procedure

The test plan according to the experimental purpose is shown in the following steps.

Step 1: Put the sensor freely into the TCB, set the temperature inside the TCB as −30 °C, and record the central wavelength of the FBGs when the temperature stabilizes.
Step 2: Increase 10 °C and test the central wavelengths after the temperature stabilizing.
Step 3: Repeat Step 2, until the temperature inside the TCB is 30 °C.
Step 4: Put the test specimen with the FBG attached as mentioned in Step 2 on the loading platform of the universal testing machine and then put them into the TCB. Adjust the temperature inside the box to 20 °C.
Step 5: Set the initial force on the specimen to 500 N, and test the central wavelength of the sensor after the temperature of the specimen stabilizes.
Step 6: The applied longitudinal force was increased to 45 kN with a step size of 5 kN. Since the initial force is 500 N, the first applied longitudinal force is 4500 N. Test the central wavelength of the two FBGs.
Step 7: Repeat Step 5 and Step 6 three times.
Step 8: Adjust the temperature inside the box to a low level (the controlled temperature during the test is −30 °C). When the temperature of the test specimen stabilizes, apply 500 N of initial force in the test direction of the specimen and then keep the displacement of the loading device unchangeable during the whole test. Then, record the central wavelength of the FBGs.
Step 9: Increase the temperature inside the TCB by 10 °C, and, after the temperature of the test specimen stabilizes, test the central wavelength of the FBGs and the corresponding pressure of the universal testing machine.
Step 10: Repeat Step 9, until the temperature inside the TCB is 30 °C.

**Figure 3:**
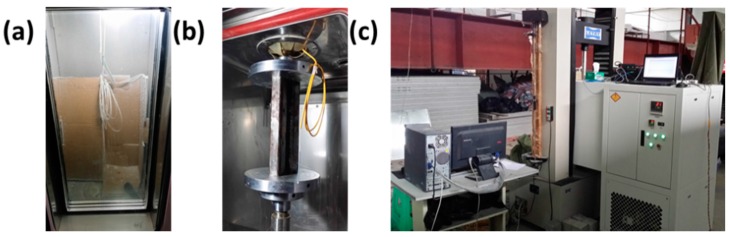
Test procedure and apparatus: (**a**) Steps 1 to 3; (**b**) Steps 4 to 10; (**c**) test apparatus.

Figure 3 shows the entire process of the indoor test and the relevant test devices. From Steps 1 to 3, the temperature sensitivity coefficients of the two FBGs can be obtained by the relationship between the temperature and the central wavelength. Additionally, *K* can be calculated by the relationship between the load and the central wavelength from Steps 4 to 7. Ultimately, a contrast verification between the theory and tested results was performed according to the data from Steps 8, 9, and 10.

### 3.3. Test Results

**Figure 4:**
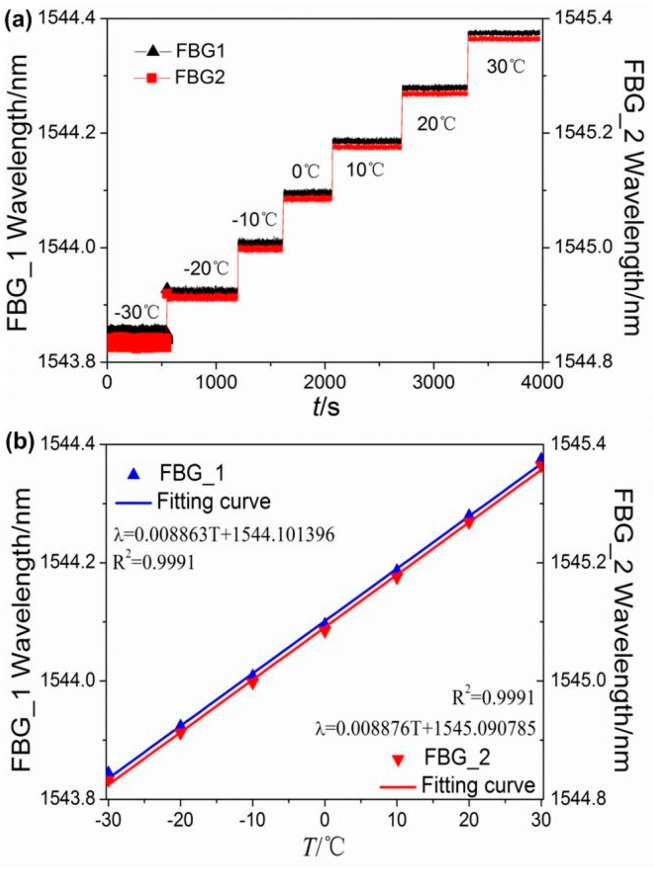
Temperature responses. (**a**) Wavelength shift as a function of temperature; (**b**) relationship between temperature variation and wavelength.

The experimental process was performed according to the plan mentioned above. Figure 4 shows the direct tested results of Steps 1, 2, and 3. The range of temperature during the test was −30 °C to +30 °C. As shown in Figure 4a, the wavelengths of both FBGs increased gradually as the temperature rose, and the central wavelength variations of the two FBGs corresponding to the two consecutive temperature steps were about 89 pm, indicating that the central wavelength variation has a good linear relation with the temperature variation. When the temperature at each step stabilized, the fluctuation of the central wavelengths of the sensors was too little to ignore, which demonstrates that the sensors have good thermal stability. The least square method could be adopted to treat the wavelength data at each temperature step; thus, the errors caused by the fluctuation of the tested data can be further reduced. Figure 4b shows the temperature characteristics of the two FBGs. The central wavelength of the FBG spectrum increased linearly as the temperature increased. From the linear fitting curve, the temperature sensitivities were 8.86 pm/°C for FBG_1 and 8.87 pm/°C for FBG_2.

**Figure 5:**
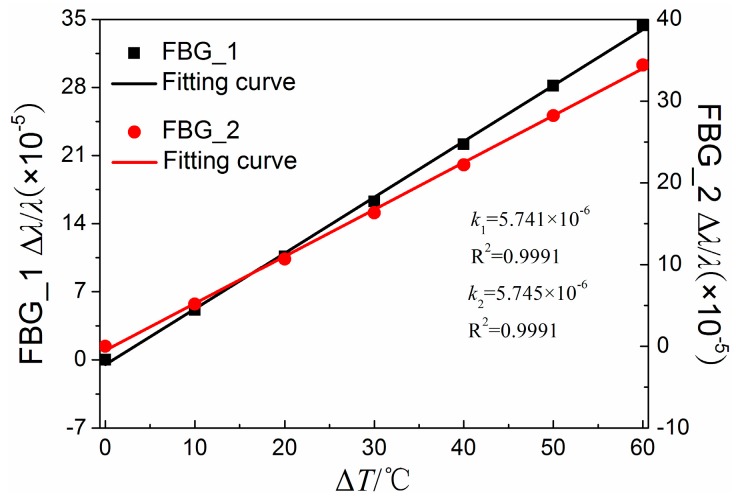
Relationship between the relative shifts of the central wavelength and the temperature variation.

Figure 5 shows the relationship between the relative shifts of the central wavelength and the temperature variation in Steps 1, 2, and 3. From the fitting results, the relative shifts of both FBGs have a good linear relation with the temperature variation. All the coefficients of determination of the fitting curve are higher than 0.999, and the slopes are 5.741 × 10^−6^ /°C and 5.745 × 10^−6^ /°C for FBG_1 and FBG_2, respectively. In addition, from Figure 5, we can observe that the difference between the temperature sensitivity coefficients of the two FBGs is less than 0.1%. Therefore, it can be assumed that the corresponding temperature sensitivity coefficients of the two FBGs are equal.

**Figure 6:**
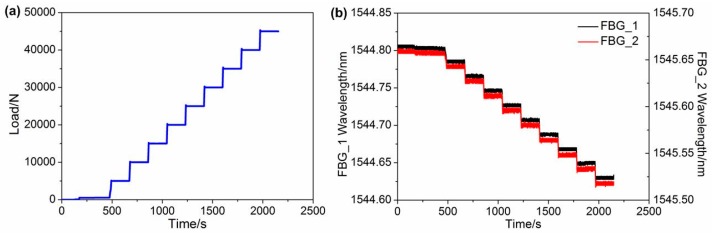
(**a**) Load variation of the universal testing machine; (**b**) real-time load response of the two FBGs.

Increasing the test time is an effective way to reduce the influence of random error on test results. Figure 6a,b shows the load variation of the universal testing machine and the real time load responses of the two FBGs, respectively. From the force load shown in Figure 6a, it can be seen that the initial force on the specimen was set to 500 N to reduce the influence of gap on relationship between load and wavelength. During the test, the temperature in the TCB was maintained at 20 °C; however, the initial central wavelength of Figure 6b is slight longer than that of Figure 4b, which is caused by the prestressed installation. Figure 6b shows that, as the applied load increases, the central wavelengths of the FBGs shift to short wavelengths.

**Figure 7:**
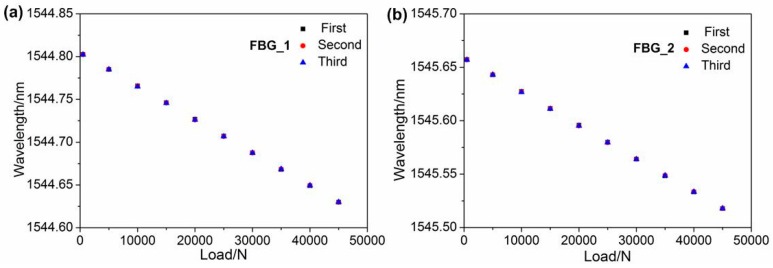
Relationship between load variation and wavelength of (**a**) FBG_1 and (**b**) FBG_2.

**Figure 8:**
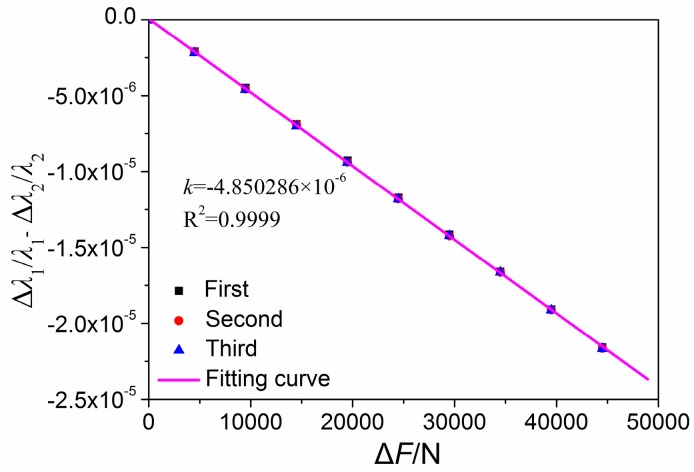
Relationship between load variation and relative wavelength shift difference.

Figure 7 presents the central wavelengths of the FBG shift versus the applied load. The central wavelengths decreased linearly as the load increased from 500 N to 45 kN. From Figure 7, we can observe that the deviation between the three processes is very small, and the maximum deviations are 0.78 pm and 0.68 pm for FBG_1 and FBG_2, respectively. Moreover, the deviations were less than 0.051%, which demonstrated that the FBGs have good measuring repeatability. Figure 8 indicates the relationship between the relative wavelength shift difference and the longitudinal additional force. The temperature in the TCB was maintained at 20 °C, since the wavelength shift induced by the temperature change and temperature terms in Equation (9) can be ignored. From Figure 8, we observe that the relationship between the relative wavelength shift difference and the longitudinal additional force is strictly linear, and the linearity is greater than 0.9999. From a linear fitting, the slope of the fitting curve is −4.850286 × 10^−6^ ε. According to Equation (10), the coefficient *K* can be obtained:

(11) $$K=kE_{s}A_{s}=0.107509$$

**Figure 9:**
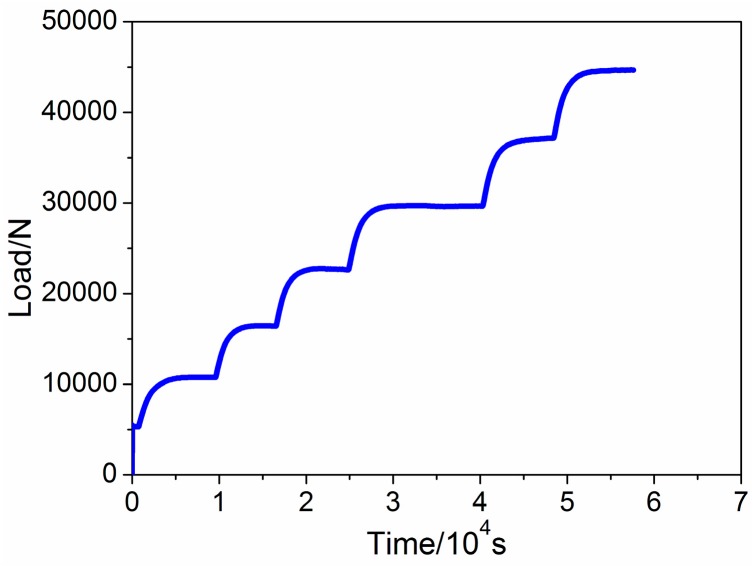
Load variation of the universal testing machine.

From Steps 8 to 10, the temperature also changed from −30 °C to 30 °C which was the same as that from Steps 1 to 3. During the temperature change, the displacement of the loading device was set as unchangeable. Figure 9 shows the load variation of the universal testing machine during the test. It can be seen that the force load of the universal testing machine increased along with the rising of the temperature in the TCB, and the corresponding force load variation was about 6571.7 N when the temperature rose by 10 °C, as shown in Figure 9. The principle of the increase in force load is the same as the mechanism that leads to a longitudinal temperature force in long seamless rails mentioned above. According to the mechanism, the corresponding force load variation should be 26,155.4 N when the temperature rises by 10 °C. Thus, the variation of the universal testing machine did not match the calculated results. This is because of the incomplete constraint of the test specimen in the test direction, such as the loading device outside the TCB and the inevitable gaps among the contact positions. By the theory of material mechanics, we can distinguish temperature force inside the rail and the emitted temperature force by strain. Moreover, the emitted temperature forces can be calculated according to the difference between the calculated result and the display of the universal testing machine.

**Figure 10:**
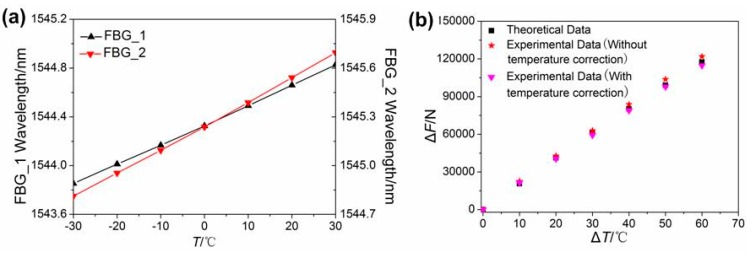
(**a**) Relationship between the central wavelength and the temperature variation; (**b**) comparison between measured and theoretical values.

Figure 10a presents the central wavelengths of the FBGs with the increasing temperature. Due to thermal expansion, the longitudinal forces will emit from the rail and cause tensile strain on the rail and sensors. Therefore, when the temperature increased, the central wavelengths of the FBGs shifted toward longer wavelengths. Based on Equations (9) and (10), the value of the parameters in the two equations, and the data in Figure 10a, the test results without and with temperature correction can be obtained, as shown in Figure 10b. The figure also contains the theoretical values. As can be seen in Figure 10b, the experimental and theoretical values are almost the same. The maximum difference between the theoretical values and the test results without and with temperature correction are 4797.1 N and 2840.8 N, which are about 4.8% and 2.4% of the theoretical values, respectively. This demonstrates that the principle of measuring the longitudinal additional force in CWR on bridges with HC-FBG sensors is correct. This also verifies that the revision with the thermal expansion of FBGs brings the results into better agreement with the theoretical data, but this benefit isn't so obvious. Thus, HC-FBG sensors is proposed as a means of measuring longitudinal additional force in CWR on bridges. In order to improve the performance of this sensor, including the sensitivity and strength enhancement, further investigation will focus on the optimization of the embedded sensors for practical applications [18,19].

## 4. Conclusions

A solution for testing the longitudinal additional force of long seamless rails on bridges is proposed. Moreover, indoor test verification is performed. Theoretical derivation results show that, in order to reduce calculation error, we should increase the difference between the ratio of the cross-sectional areas and the ratio of the strain sensitivity coefficient. Additionally, the HC-FBGs are inscribed in a segment single-mode fiber, so the difference of the temperature sensitivity coefficient is insignificant. Experimental results show that the maximum difference between the theoretical values and the test results without and with temperature correction are about 4.8% and 2.4% of the theoretical values, respectively. In order to simplify the measuring process in engineering applications, the influence of the temperature can be ignored. Experimental results also proved the credibility of the proposed test principle of HC-FBG sensors by theoretical analysis. The next step is to design a proper package of the HC-FBG sensor and run the field test to measure the longitudinal additional force in CWR on bridges.

## References

[1] Ahmad S.S., Mandal N.K., Chattopadhyay G., Powell J. (2013). Development of a unified railway track stability management tool to enhance track safety. J. Rail Rapid Transit.

[2] Kish A., Mcwilliams R.S., Harrison H. Track buckling hazard detection and rail stress management. Proceedings of the 9th World Conference on Railway Research.

[3] Kumar S., Gupta S., Ghodrati B., Kumar U. (2010). An approach for risk assessment of rail defects. Int. J. Reliab. Qual. Saf. Eng..

[4] Tam H.Y., Lee T., Ho S.L., Haber T., Graver T., Méndez A. (2007). Utilization of fiber optic Bragg grating sensing systems for health monitoring in railway applications. Struct. Health Monit..

[5] Kuen L.K., Lee T.K., Ho S.L., Chan H.K. (2006). Structural integrity studies of the body shells of light rail cars. HKIE Trans. Hong Kong Inst. Eng..

[6] Lee K.Y., Lee K.K., Ho S.L. (2004). Exploration of using FBG sensor for derailment detector. WSEAS Trans. Top. Syst..

[7] Kang D., Chung W. (2009). Integrated monitoring scheme for a maglev guide way using multiplexed FBG sensor arrays. NDT&E Int..

[8] Wang S.G., Yan L.S., Pan W., Luo B., Guo L.K., Wen K.H. (2011). Investigation of track sensor based on matched fiber Bragg gratings. J. China Railw. Soc..

[9] Zhang Z., Yan L., Pan W., Luo B., Wang P., Guo L., Zhou W. (2012). Sensitivity enhancement of strain sensing utilizing a differential pair of fiber Bragg gratings. Sensors.

[10] Kouroussis G., Caucheteur C., Kinet D., Alexandrou G., Verlinden O., Moeyaert V. (2015). Review of trackside monitoring solutions: From strain gages to optical fibre sensors. Sensors.

[11] Yan L., Zhang Z., Wang P., Pan W., Guo L., Luo B., Wen K., Wang S., Zhao G. (2011). Fiber sensors for strain measurements and axle-counting in high-speed railway applications. IEEE Sens. J..

[12] Wei C., Lai C., Liu S., Chung W.H., Ho T.K., Tam H., Ho S.L., McCusker A., Kam J., Lee K.Y. (2010). A Fiber Bragg Grating Sensor System for Train Axle Counting. IEEE Sens. J..

[13] Catalano A., Bruno F.A., Pisco M., Cutolo A., Cusano A. (2014). An intrusion detection system for the protection of railway assets using fber Bragg grating sensors. Sensors.

[14] Buggy S.J., James S.W., Staines S., Carroll R., Kitson P., Farrington D. (2016). Railway track component condition monitoring using optical fibre Bragg grating sensors. Meas. Sci. Technol..

[15] Wang P., Xie K.Z., Shao L.Y., Yan L., Xu J., Chen R. (2016). Longitudinal force measurement in continuous welded rail with bi-directional FBG strain sensors. Smart Mater. Struct..

[16] Dai X. (2013). Research on Methods and Key Technologies on High-Speed Track Structure Monitoring Based on Fiber Grating. Ph.D. Thesis.

[17] Sun X.F., Fang X.S., Guan L.T. (2002). Mechanics of Materials.

[18] Leduc D., Lecieux Y., Morvan P.A., Lupi C. (2013). Architecture of optical fiber sensor for the simultaneous measurement of axial and radial strains. Smart Mater. Struct..

[19] Guyard R., Leduc D., Lecieux Y., Lupi C. (2016). Superposition of fiber Bragg and LPG gratings for embedded strain measurement. Comptes Rendus Phys..

